# Supplementary material for: Cell-specific proteome analyses of human bone marrow reveal molecular features of age-dependent functional decline
Source: Nat Commun. 2018 Oct 1;9:4004. doi: 10.1038/s41467-018-06353-4 (PMC6167374; doi:10.1038/s41467-018-06353-4)
Supplement: Supplementary file 1 — Supplementary Information [file 41467_2018_6353_MOESM1_ESM.pdf]

Cell-specific proteome analyses of human bone marrow reveal molecular features of age-dependent functional decline

Marco L. Hennrich[1,2,*], Natalie Romanov[1,*], Patrick Horn[2,3,*], Samira Jaeger[4], Volker Eckstein[3], Violetta Steeples[5], Fei Ye[1,2], Ximing Ding[1,2,3], Laura Poisa-Beiro[2,3], Mang Ching Lai[1,2], Benjamin Lang[1], Jacqueline Boultwood[5], Thomas Luft[3], Judith B. Zaugg[1,2], Andrea Pellagatti[5], Peer Bork[1,2,6], Patrick Aloy[4,7], Anne-Claude Gavin[1,2,#], Anthony D. Ho[2,3,#]

Affiliations:

[1] European Molecular Biology Laboratory (EMBL), Structural and Computational Biology Unit, Meyerhofstrasse 1, D69117 Heidelberg, Germany.

[2] Molecular Medicine Partnership Unit (MMPU), Meyerhofstrasse 1, D69117 Heidelberg, Germany.

[3] Department of Medicine V, Heidelberg University, D69120 Heidelberg, Germany.

[4] Institute for Research in Biomedicine (IRB), the Barcelona Institute of Science and Technology, 08028 Barcelona, Catalonia, Spain.

[5] Radcliffe Department of Medicine, University of Oxford, and Oxford BRC Haematology Theme, OX3 9DU Oxford, UK.

[6] Department of Bioinformatics, Biocenter, University of Würzburg, D97074 Würzburg, Germany.

[7] Institució catalane de Recerca i Estudis Avançats (ICREA), 08010 Barcelona, Catalonia, Spain.

* Contributed equally

[#] Corresponding authors: Anthony D. Ho (anthony_dick.ho@urz.uni-heidelberg.de) and Anne-Claude Gavin (gavin@embl.de)

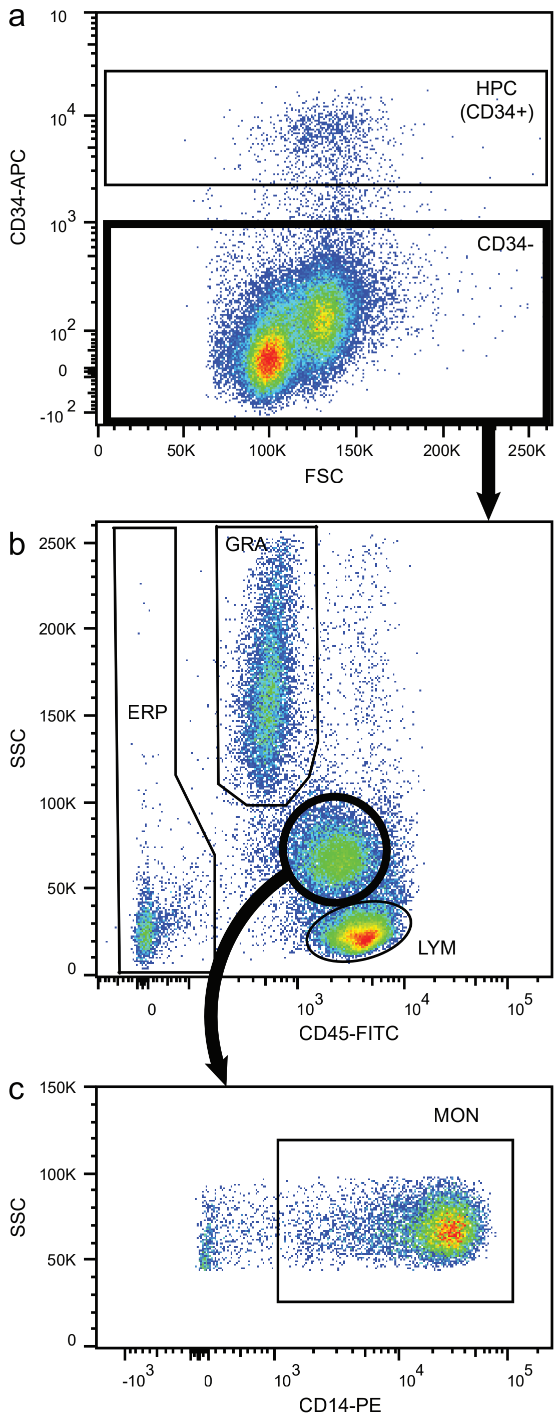

Supplementary Figure 1: Example of the FACS gating strategy for the isolation of the different cell populations. (a) Cells are sorted based on their CD34 expression. HPCs are defined as CD34 positive cells. (b) CD34 negative cells are further gated by the side scatter and their CD45 expression. ERPs are defined as CD45 negative, GRAs as CD45 medium and SSC high, and LYMs as CD45 high and SSC low. (c) MONs (SSC med; CD45 high) are further isolated based on their CD14 expression.

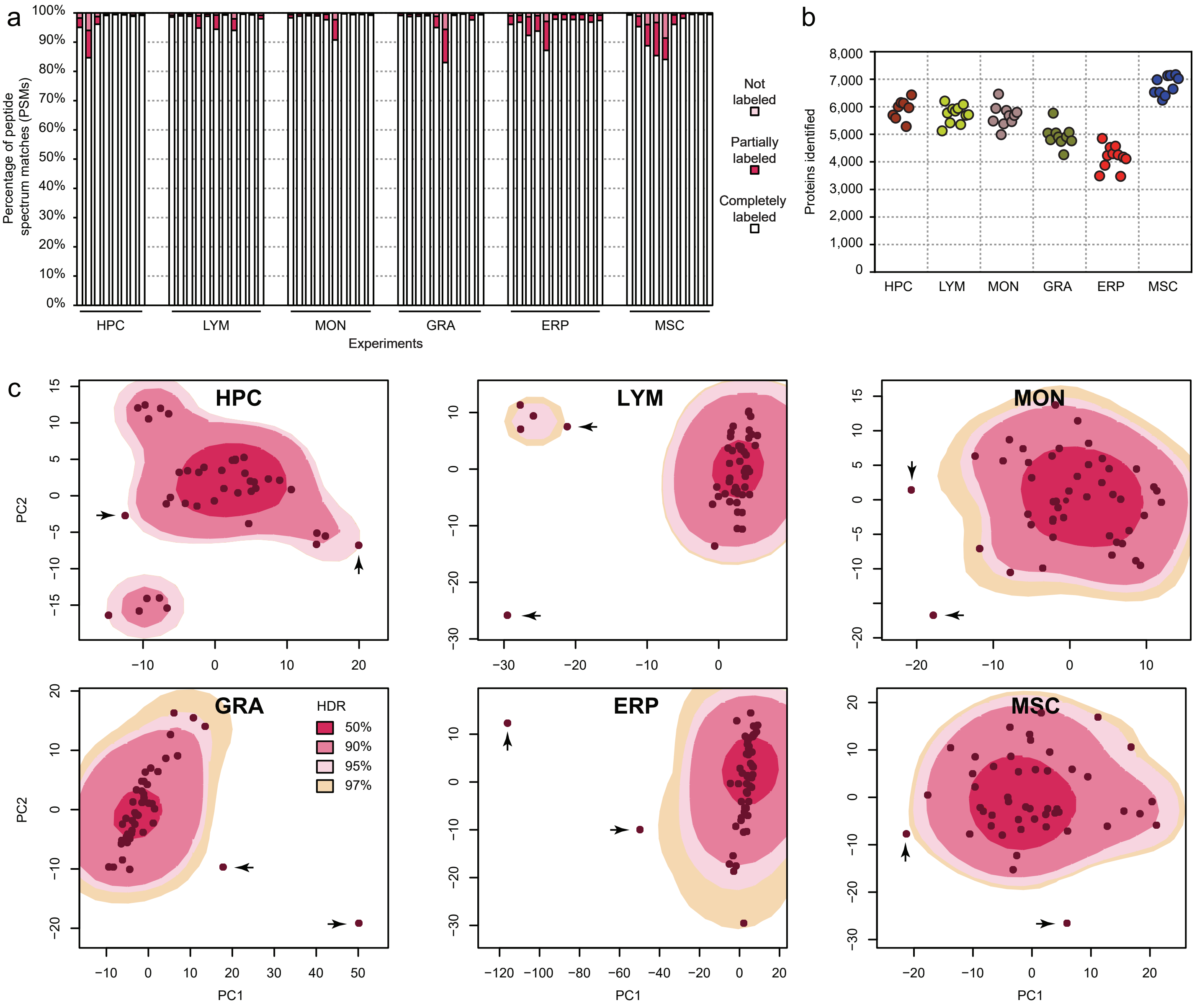

Supplementary Figure 2: Labeling efficiency, protein number and outlier analysis as part of the quality control analysis. (a) Completely labeled, partially labeled and unlabeled peptide spectrum matches (PSMs) expressed as a percentage of all PSMs are stacked. (b) Protein identification number for all experiments. Each point indicates the number of proteins identified in a TMT-6-plex experiment. (c) A principal component analysis (PCA) was performed on the log2-transformed data and the first two principle components (PC1 and PC2) were plotted against each other. Highest density regions (HDR plot) of 50, 90, 95 and 97 percent probability were visualized based on Hyndman[1] and samples with >97% probability were defined as outliers to be discarded. The discarded samples are marked by arrows.

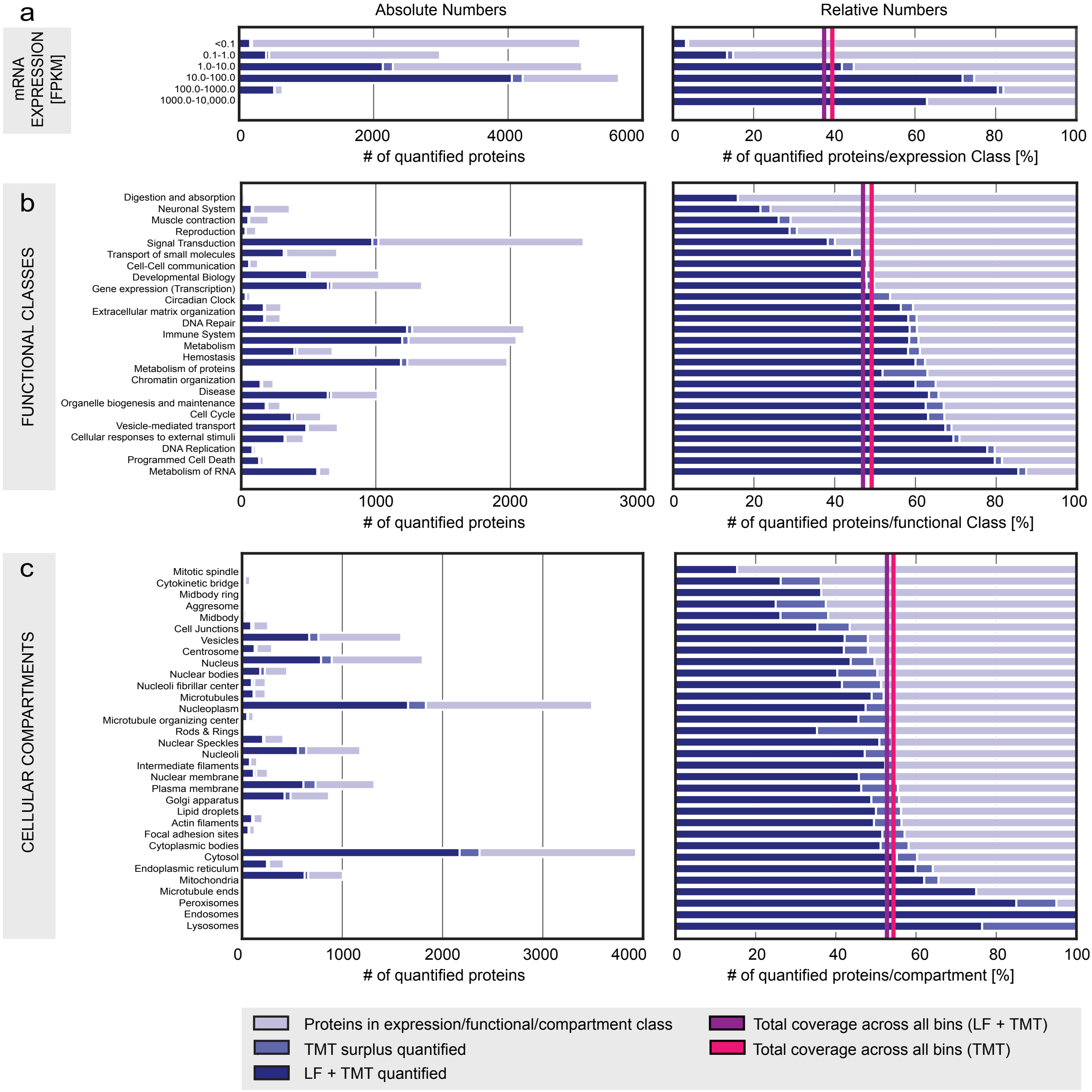

Supplementary Figure 3: Overview of the mRNA expression levels, functional classes, as well as cellular compartments of all quantified proteins in comparison with the total human proteome. (a) Label-free (LF) and TMT quantified proteins are grouped by their mRNA expression levels and their absolute and relative number are visualized as fraction of the total human proteome. The expression classes are defined according to transcript levels measured in the bone marrow tissue by Uhlen *et al.* (2015)[2]. (b + c) The quantified proteins are grouped by function (b) and cellular compartment (c). The functional classes are defined by the highest hierarchical level of the Reactome pathway database [http://www.reactome.org] and the compartment classes are defined according to the Human Protein Atlas[2]. The red and violet vertical lines illustrate the total coverages of the quantified proteins across all bins in the presented class.

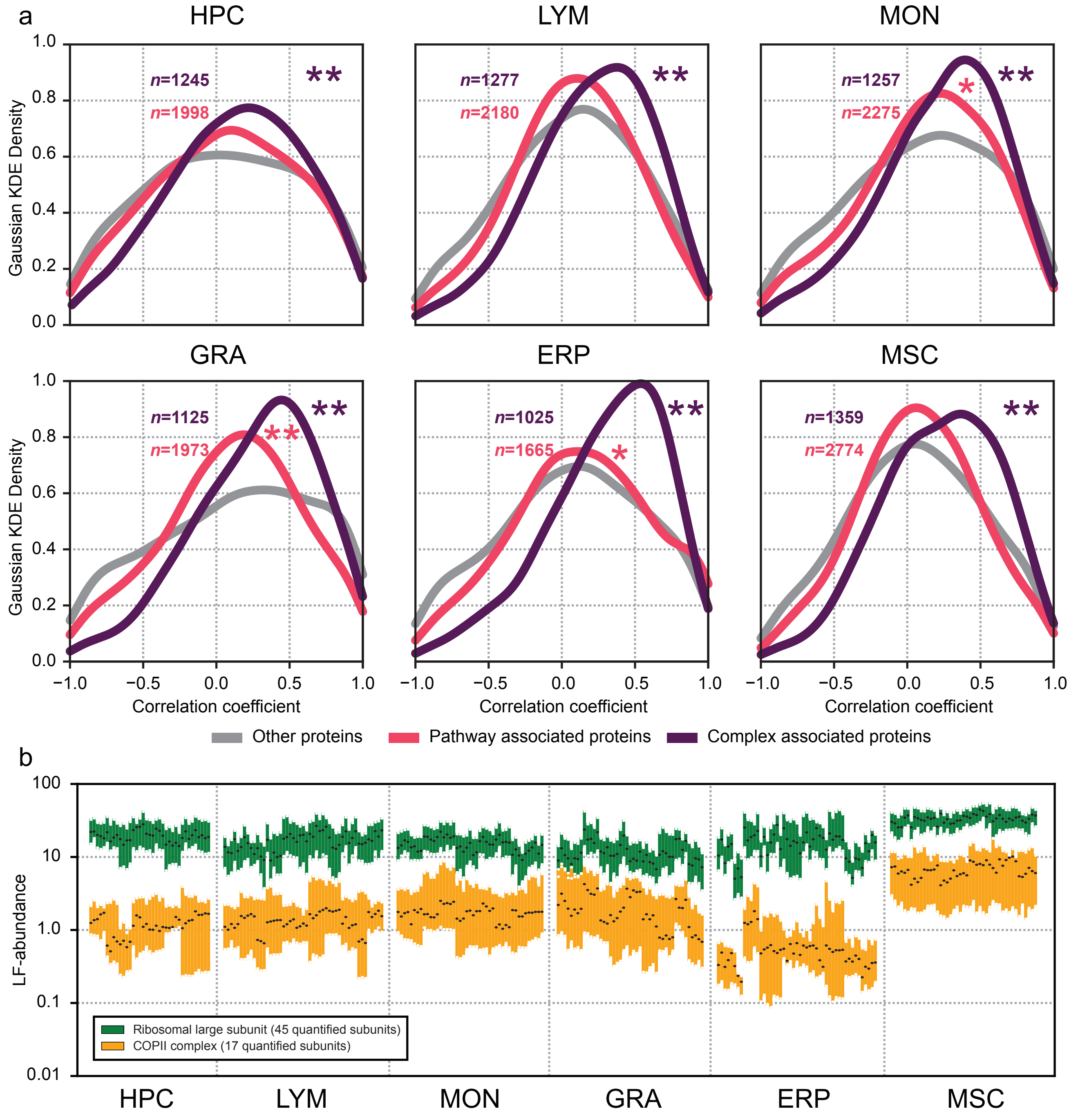

Supplementary Figure 4: Abundance of proteins within complexes and pathways correlate better across donors compared to proteins that do not form complexes or are not part of a pathway. (a) Density plots for each cell population visualizing the co-variation of proteins within a complex or pathway. Proteins within a complex (purple) have a clear shift towards higher correlation compared to proteins that are not annotated to a complex or pathway (other proteins). *P*-values were calculated using random sampling of the distributions and calculating the Kolmogorov-Smirnov test statistic 1,000 times to then estimate the median *p*-value. Stars indicate the significance value with (*) equaling a *p*-value < 0.05 and (**) a *p*-value < 0.01. The total number of proteins associated to complexes, and pathways in each cell population, is presented in each subplot in the respective colour (*n*). (b) For each of the 270 samples across all cell populations the label-free (LF) abundances of proteins, belonging to the large ribosomal subunit (green) or the COPII complex (orange), are summarized in box plots. The central line in the box plots indicates the median, and the bottom and top edges of the box the interquartile range (IQR). No whiskers are shown.

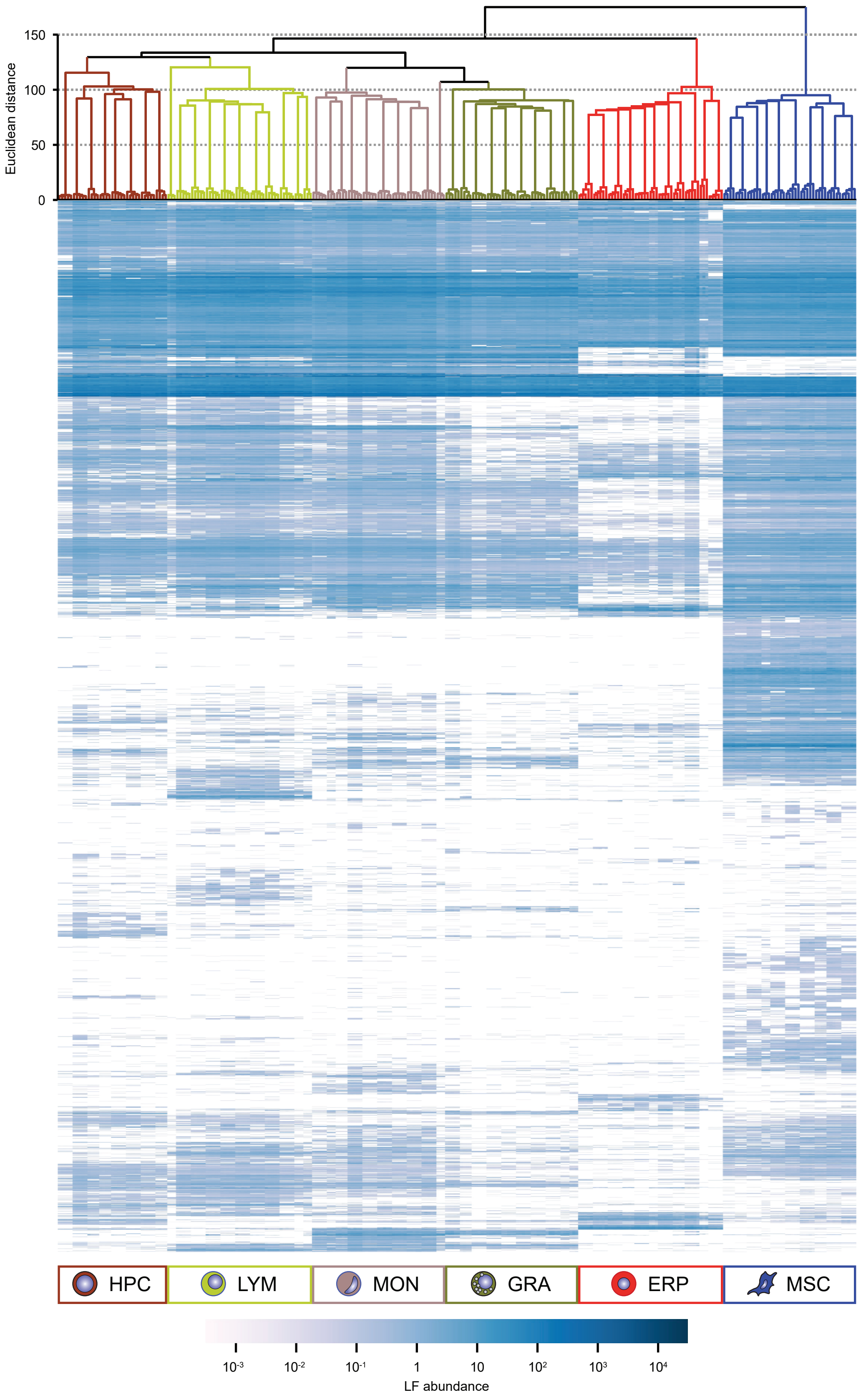

Supplementary Figure 5: Hierarchical clustering of all 270 samples quantified by label-free (LF) quantification. The LF abundance (blue colour scale) of all proteins from all samples was used for clustering using Euclidean distances. White colouring defines absence of a quantification event. The samples cluster according to their cell population.

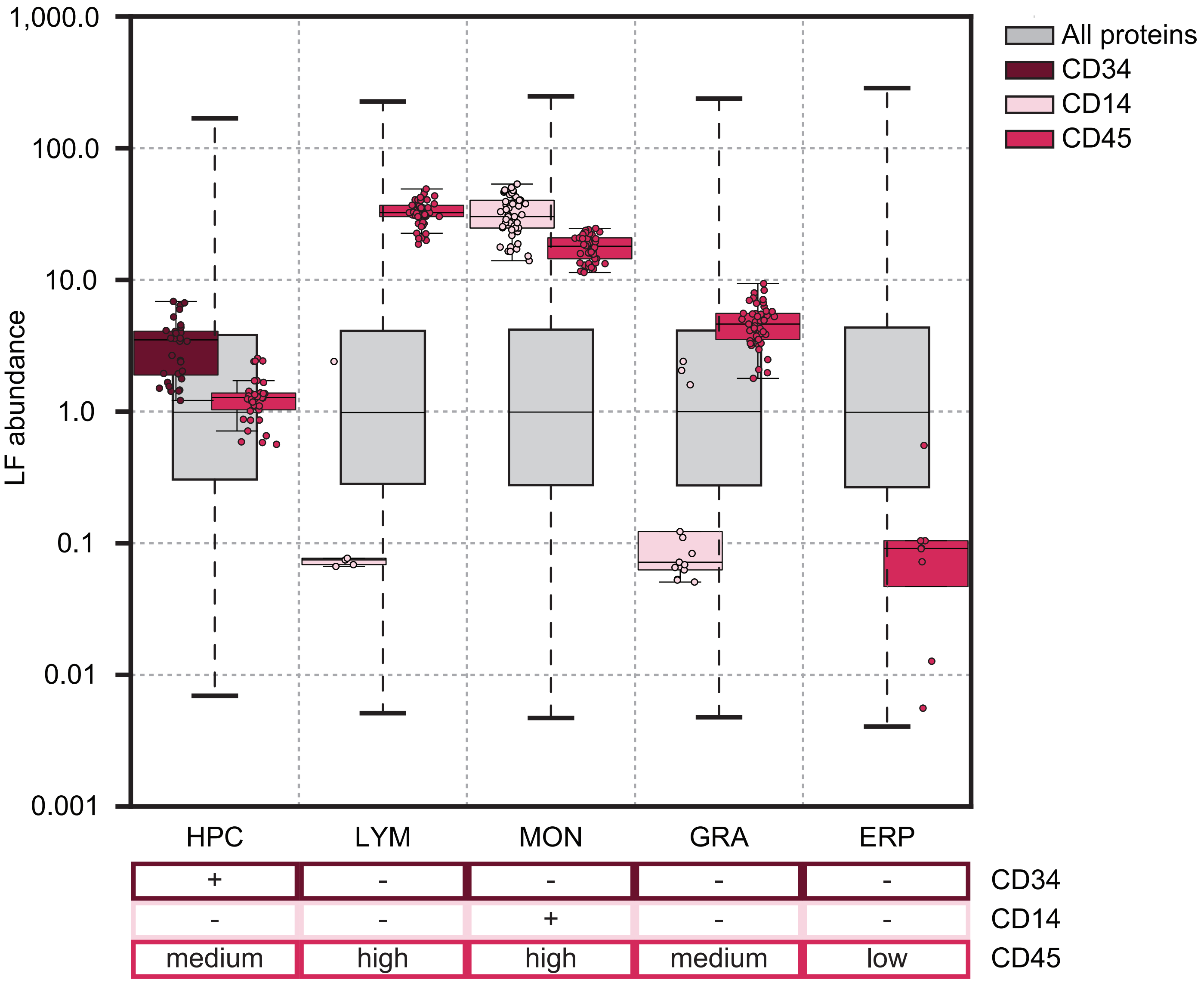

Supplementary Figure 6: Visualization of the relative label-free (LF) abundance of surface markers used in FACS for sorting the cell populations. The dots represent the results of the individual samples, if the protein was quantified. Protein abundance (top) correlates well with gates (bottom) used for FACS with CD34 being specific for HPC, LYM and MON being CD45^{+} (PTPRC), GRA being CD45med, ERP being CD45^{-}, and MON being gated for CD14^{+}. For comparison the results of all quantified proteins are represented as a grey box plot for each individual cell population. The central line in the box plots indicates the median, the bottom and top edges of the box the interquartile range (IQR), and the box plot whiskers represent 1.5 times the IQR.

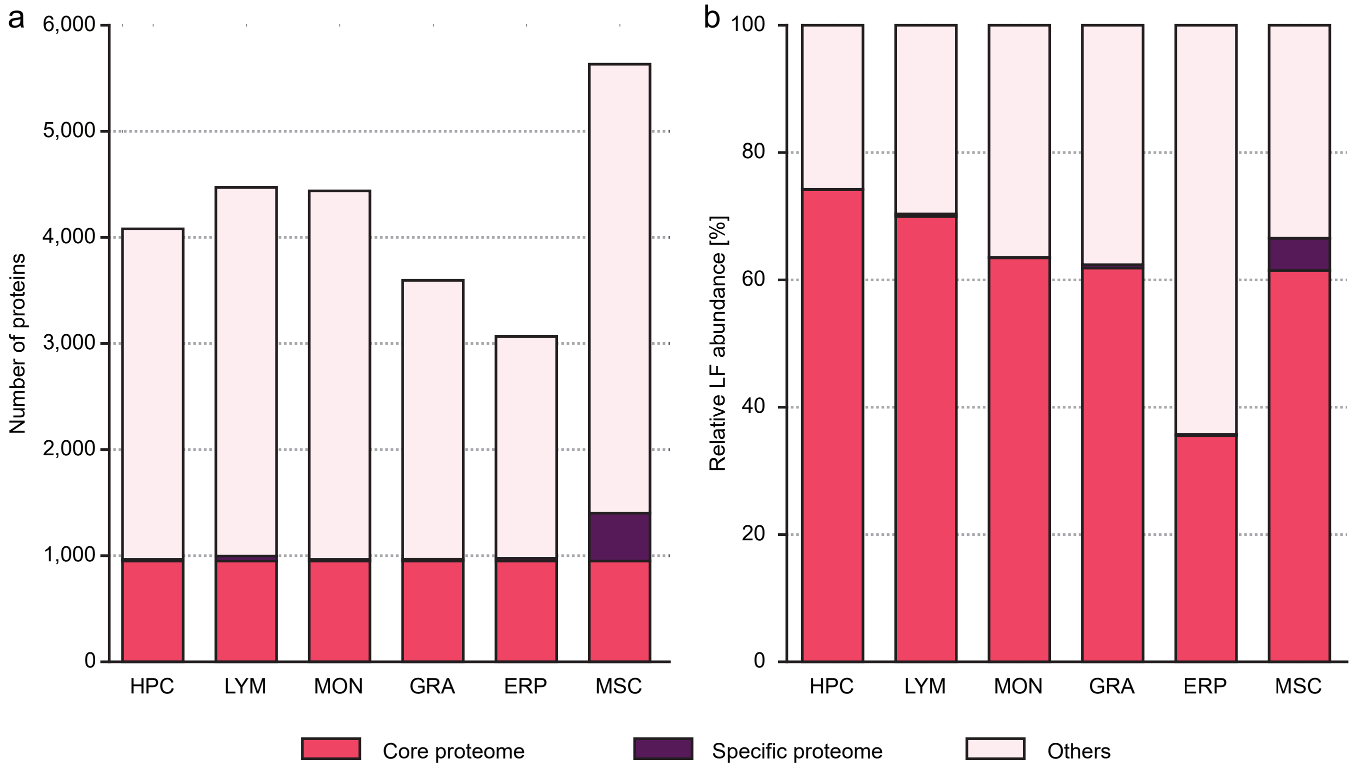

Supplementary Figure 7: Characterization of the number and relative abundance of the core and specific proteome in the individual cell populations. (a) Overview on the number of proteins quantified by label-free quantification in each cell population. Colours indicate the fraction of proteins corresponding to the core proteome (≥ 85% sample coverage in all cell populations) or specific proteome (≥ 85% sample coverage in a single cell population and < 15% sample coverage in all other cell populations), and remainder (others). (b) Overview on the relative label-free (LF) abundances of the corresponding proteome categories in each cell population relative to the sum of the abundances of all proteins.

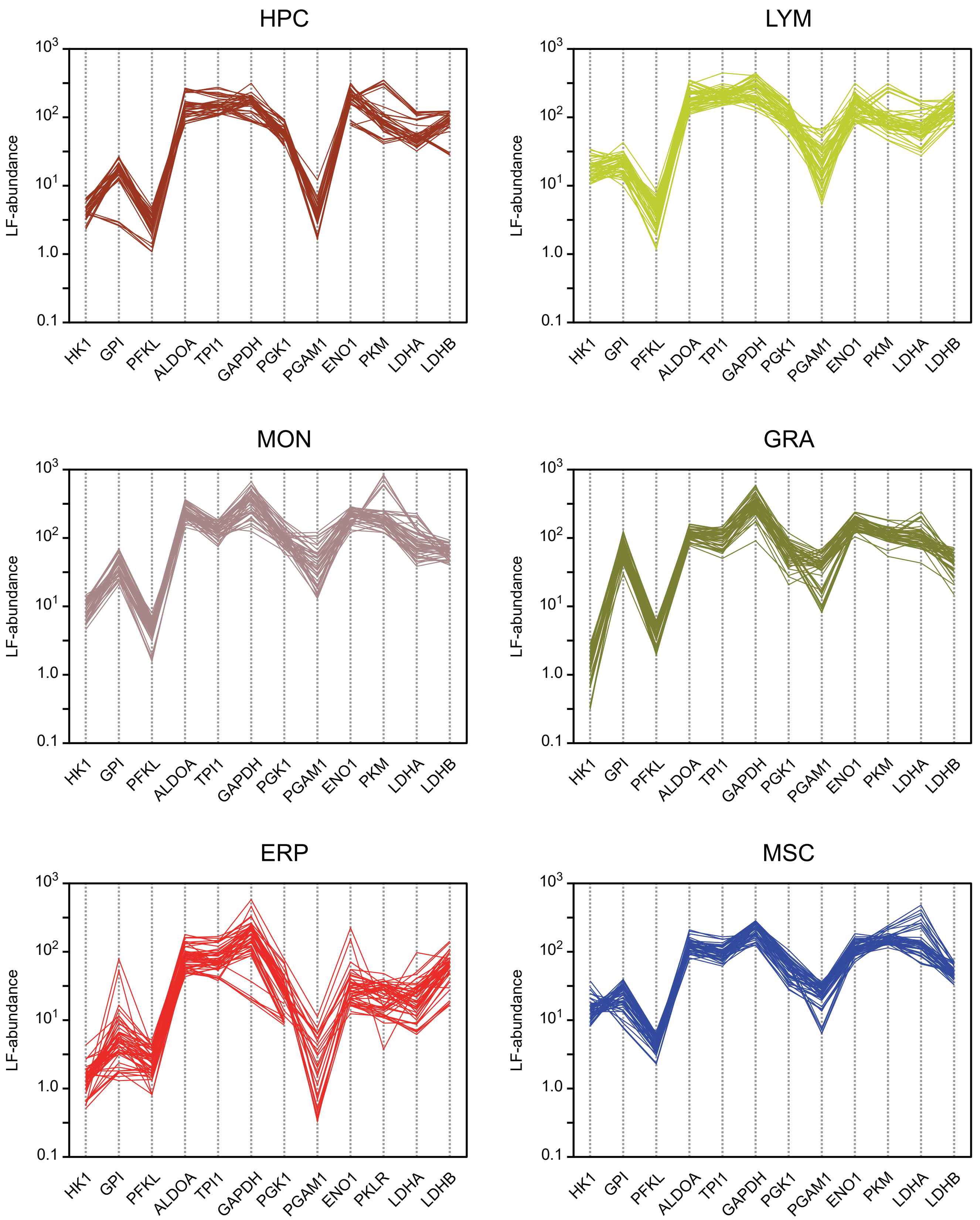

Supplementary Figure 8. The stoichiometry of proteins of the glycolytic pathway are maintained across donors in the different cell populations. Each line connects the label-free (LF) abundances of the major proteins of glycolysis for one individual donor. The glycolytic enzymes are annotated at the x-axis and each subplot represent the results for one of the six different cell populations.

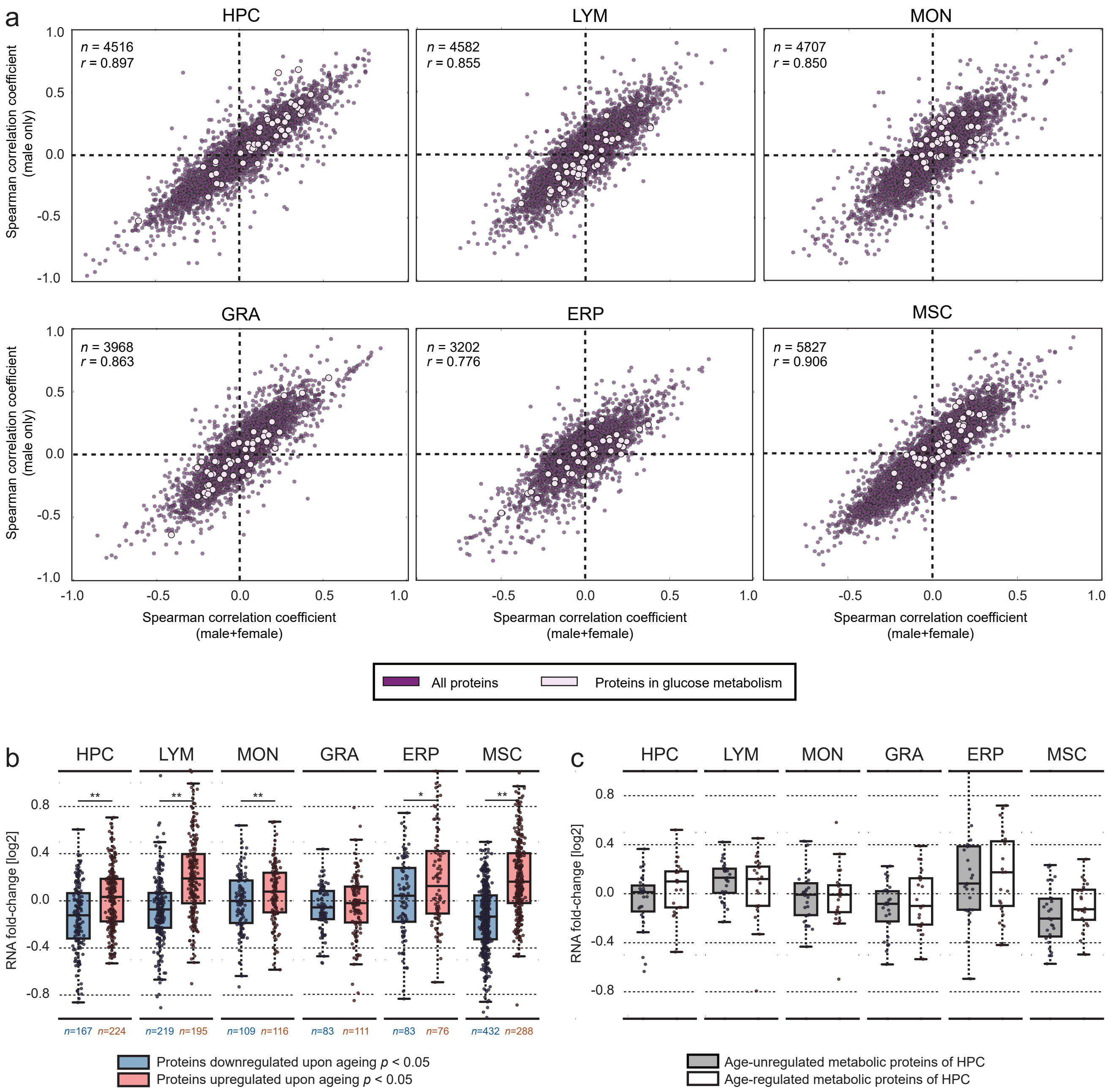

Supplementary Figure 9. Overview on gender effects and transcript expression changes upon ageing. (a) The Spearman correlation coefficients for protein changes upon ageing are calculated for all samples and for only male samples per each cell population. The correlation coefficients of protein changes of all samples (x-axis) are plotted against the male only correlation coefficients (y-axis). Proteins that are related to the glucose metabolism are highlighted as grey dots, and all other (purple) dots represent all other quantified proteins (*n* is given in the upper left corner, respectively). (b) The box-plots depict RNA fold-changes of proteins that are down-regulated (blue) or up-regulated (red) with age according to the proteomics data (p-value<0.05). Fold-changes were calculated between old (>50 years) and young (<30 years) donors (Mann-Whitney U-test: p-value < 0.01 (**), p-value < 0.05 (*)). (c) The box-plots depict RNA fold-changes of glycolytic and TCA-related proteins that are not altered upon ageing in HPC (blue) and that are altered upon ageing (red). Proteins from Figure 4a+c were taken into account. Significance was assessed using a Mann-Whitney U-test (p-value for HPC = 0.052). (b+c) The central line in the box plots indicates the median, the bottom and top edges of the box the IQR, and the box plot whiskers represent 1.5 times the IQR.

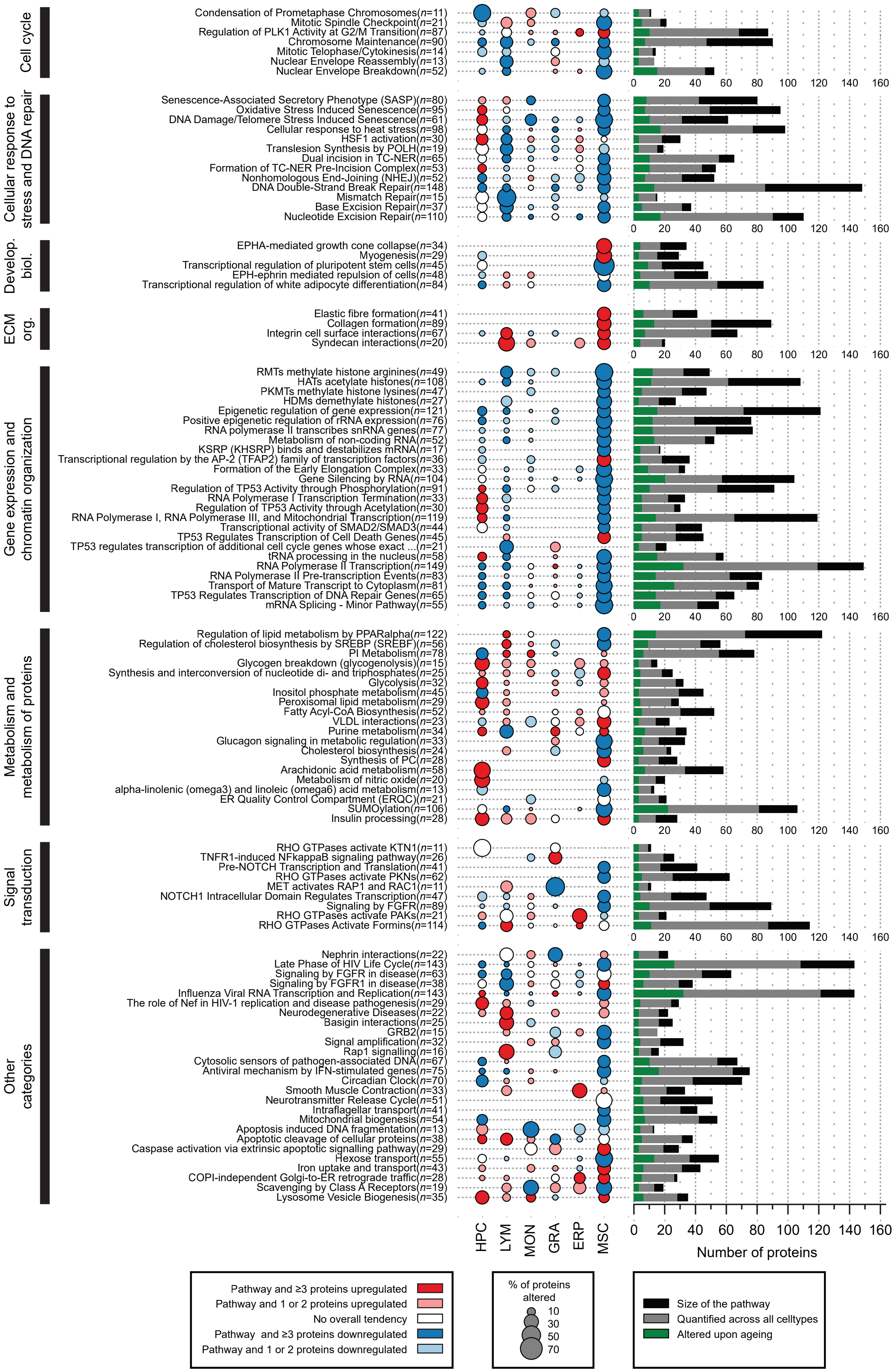

Cell cycle
Condensation of Prometaphase Chromosomes(n=11)
Mitotic Spindle Checkpoint(n=21)
Regulation of PLK1 Activity at G2/M Transition(n=87)
Chromosome Maintenance(n=90)
Mitotic Telophase/Cytokinesis(n=14)
Nuclear Envelope Reassembly(n=13)
Nuclear Envelope Breakdown(n=52)
Cellular response to stress and DNA repair
Senescence-Associated Secretory Phenotype (SASP)(n=80)
Oxidative Stress Induced Senescence(n=95)
DNA Damage/Telomere Stress Induced Senescence(n=61)
Cellular response to heat stress(n=98)
HSF1 activation(n=30)
Translesion Synthesis by POLH(n=19)
Dual incision in TC-NER(n=65)
Formation of TC-NER Pre-Incision Complex(n=53)
Nonhomologous End-Joining (NHEJ)(n=52)
DNA Double-Strand Break Repair(n=148)
Mismatch Repair(n=15)
Base Excision Repair(n=37)
Nucleotide Excision Repair(n=110)
Develop. biol.
EPHA-mediated growth cone collapse(n=34)
Myogenesis(n=29)
Transcriptional regulation of pluripotent stem cells(n=45)
EPH-ephrin mediated repulsion of cells(n=48)
Transcriptional regulation of white adipocyte differentiation(n=84)
ECM org.
Elastic fibre formation(n=41)
Collagen formation(n=89)
Integrin cell surface interactions(n=67)
Syndecan interactions(n=20)
Gene expression and chromatin organization
RMTs methylate histone arginines(n=49)
HATs acetylate histones(n=108)
PKMTs methylate histone lysines(n=47)
HDMs demethylate histones(n=27)
Epigenetic regulation of gene expression(n=121)
Positive epigenetic regulation of rRNA expression(n=76)
RNA polymerase II transcribes snRNA genes(n=77)
Metabolism of non-coding RNA(n=52)
KSRP (KHSRP) binds and destabilizes mRNA(n=17)
Transcriptional regulation by the AP-2 (TFAP2) family of transcription factors(n=36)
Formation of the Early Elongation Complex(n=33)
Gene Silencing by RNA(n=104)
Regulation of TP53 Activity through Phosphorylation(n=91)
RNA Polymerase I Transcription Termination(n=33)
Regulation of TP53 Activity through Acetylation(n=30)
RNA Polymerase I, RNA Polymerase III, and Mitochondrial Transcription(n=119)
Transcriptional activity of SMAD2/SMAD3(n=44)
TP53 Regulates Transcription of Cell Death Genes(n=45)
TP53 regulates transcription of additional cell cycle genes whose exact ...(n=21)
tRNA processing in the nucleus(n=58)
RNA Polymerase II Transcription(n=149)
RNA Polymerase II Pre-transcription Events(n=83)
Transport of Mature Transcript to Cytoplasm(n=81)
TP53 Regulates Transcription of DNA Repair Genes(n=65)
mRNA Splicing - Minor Pathway(n=55)
Metabolism and metabolism of proteins
Regulation of lipid metabolism by PPARalpha(n=122)
Regulation of cholesterol biosynthesis by SREBP (SREBF)(n=56)
PI Metabolism(n=78)
Glycogen breakdown (glycogenolysis)(n=15)
Synthesis and interconversion of nucleotide di- and triphosphates(n=25)
Glycolysis(n=32)
Inositol phosphate metabolism(n=45)
Peroxisomal lipid metabolism(n=29)
Fatty Acyl-CoA Biosynthesis(n=52)
VLDL interactions(n=23)
Purine metabolism(n=34)
Glucagon signaling in metabolic regulation(n=33)
Cholesterol biosynthesis(n=24)
Synthesis of PC(n=28)
Arachidonic acid metabolism(n=58)
Metabolism of nitric oxide(n=20)
alpha-linolenic (omega3) and linoleic (omega6) acid metabolism(n=13)
ER Quality Control Compartment (ERQC)(n=21)
SUMOylation(n=106)
Insulin processing(n=28)
Signal transduction
RHO GTPases activate KTN1(n=11)
TNFR1-induced NFkappaB signaling pathway(n=26)
Pre-NOTCH Transcription and Translation(n=41)
RHO GTPases activate PKNs(n=62)
MET activates RAP1 and RAC1(n=11)
NOTCH1 Intracellular Domain Regulates Transcription(n=47)
Signaling by FGFR(n=89)
RHO GTPases activate PAKs(n=21)
RHO GTPases Activate Formins(n=114)
Other categories
Nephrin interactions(n=22)
Late Phase of HIV Life Cycle(n=143)
Signaling by FGFR in disease(n=63)
Signaling by FGFR1 in disease(n=38)
Influenza Viral RNA Transcription and Replication(n=143)
The role of Nef in HIV-1 replication and disease pathogenesis(n=29)
Neurodegenerative Diseases(n=22)
Basigin interactions(n=25)
GRB2(n=15)
Signal amplification(n=32)
Rap1 signalling(n=16)
Cytosolic sensors of pathogen-associated DNA(n=67)
Antiviral mechanism by IFN-stimulated genes(n=75)
Circadian Clock(n=70)
Smooth Muscle Contraction(n=33)
Neurotransmitter Release Cycle(n=51)
Intraflagellar transport(n=41)
Mitochondrial biogenesis(n=54)
Apoptosis induced DNA fragmentation(n=13)
Apoptotic cleavage of cellular proteins(n=38)
Caspase activation via extrinsic apoptotic signalling pathway(n=29)
Hexose transport(n=55)
Iron uptake and transport(n=43)
COPI-independent Golgi-to-ER retrograde traffic(n=28)
Scavenging by Class A Receptors(n=19)
Lysosome Vesicle Biogenesis(n=35)
HPC
LYM
MON
GRA
ERP
MSC
0 20 40 60 80 100 120 140 160
Number of proteins
Pathway and ≥3 proteins upregulated
Pathway and 1 or 2 proteins upregulated
No overall tendency
Pathway and ≥3 proteins downregulated
Pathway and 1 or 2 proteins downregulated
% of proteins altered
10
30
50
70
Size of the pathway
Quantified across all celltypes
Altered upon ageing

Supplementary Figure 10: Age-affected pathways in the individual cell populations. (a) A selection of pathways from the Reactome database that show prominent changes upon ageing are depicted. Pathways were required to have between 5 and 150 members, to be sufficiently covered in at least one cell population (> 30% of the proteins are quantified), and to have at least 20% of its quantified components being significantly (*p*-value < 0.05, Spearman correlation) altered upon ageing. The area of the bubbles represents the percentage of proteins quantified by TMT that are significantly (*p*-value < 0.05, Spearman correlation) altered. If no bubble is shown, no protein of the pathway has been observed to be significantly altered in the respective cell population or no protein of the pathway has been quantified. The colour of the bubbles codes for the direction of the alteration, with red indicating an overall increase of the pathway members with at least three proteins being upregulated and pink an overall increase with one or two proteins being upregulated. Blue codes for pathways with an overall trend towards downregulation, with strong blue coding for pathways with at least three proteins being downregulated and light blue containing one or two proteins being downregulated. The colour white indicates that no overall tendency for the proteins associated with the corresponding pathway could be observed. The bars on the right-hand side of each pathway illustrate the number of proteins being significantly altered upon ageing regardless of the cell population (green), being quantified by TMT (grey), and the total number of members of the pathway (black) as also mentioned in the pathway annotation (*n*). The grouping of the pathways on the left side is based on the highest hierarchy levels defined in Reactome, e.g. extracellular matrix organisation (ECM org.) and developmental biology (develop. biol.).

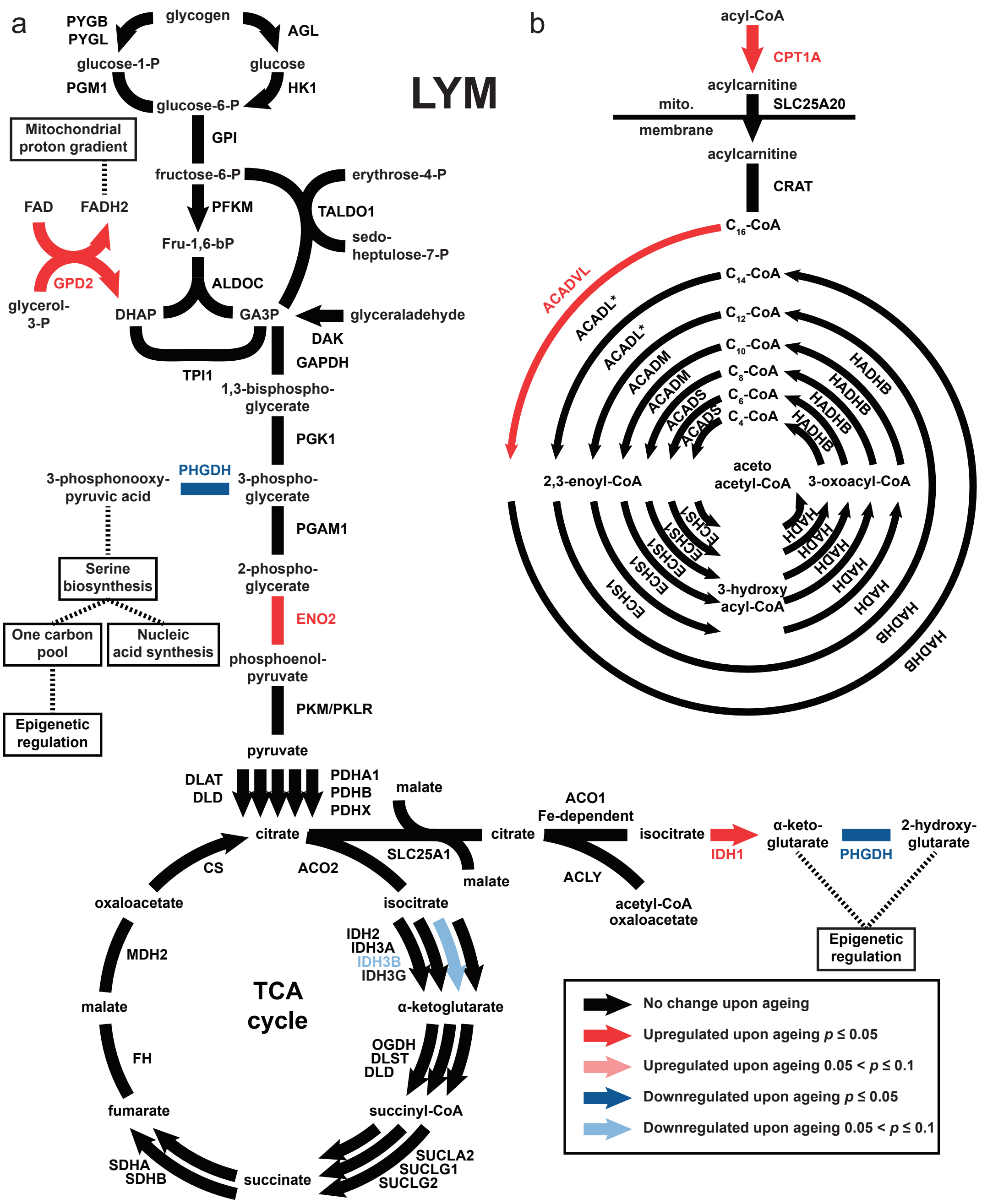

Supplementary Figure 11 a-b: Prominent changes upon ageing in the central carbon metabolism of LYM. (a) Glucose metabolism and the tricarboxylic acid (TCA) cycle are illustrated with arrows representing unidirectional reactions and strokes representing bidirectional reactions. The gene names of the respective enzymes are written in capital letters and the colour encodes changes upon ageing, as described in the legend. (b) The mitochondrial beta-oxidation of saturated fatty acids is depicted as described for (a). A star indicates that the particular protein was not covered for quantification. All *p*-values (*p*) are based on Spearman correlation analyses.

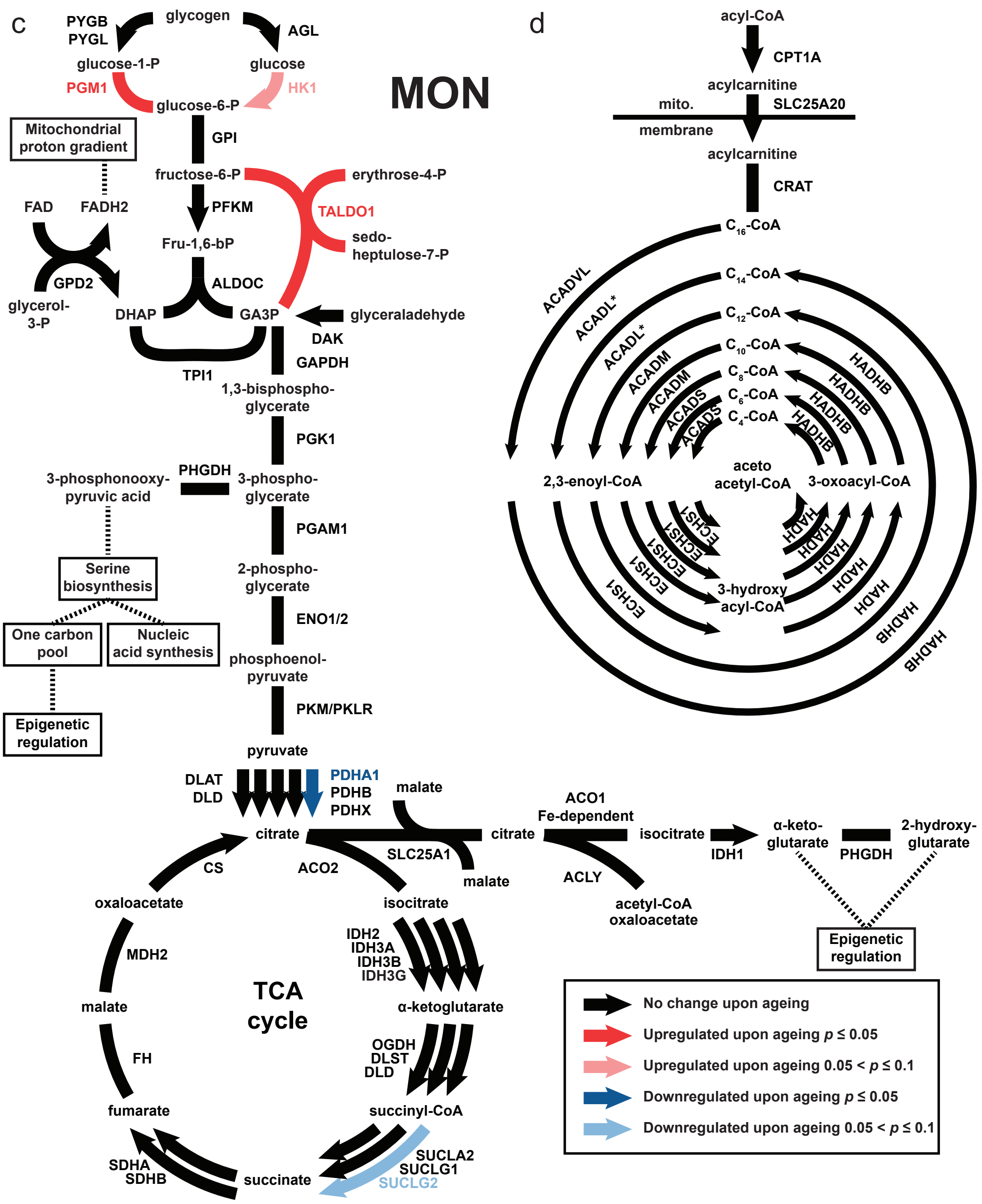

Supplementary Figure 11 c-d: Prominent changes upon ageing in the central carbon metabolism of MON. (c) Glucose metabolism and the tricarboxylic acid (TCA) cycle are illustrated with arrows representing unidirectional reactions and strokes representing bidirectional reactions. The gene names of the respective enzymes are written in capital letters and the colour encodes changes upon ageing, as described in the legend. (d) The mitochondrial beta-oxidation of saturated fatty acids is depicted as described for (c). A star indicates that the particular protein was not covered for quantification. All *p*-values (*p*) are based on Spearman correlation analyses.

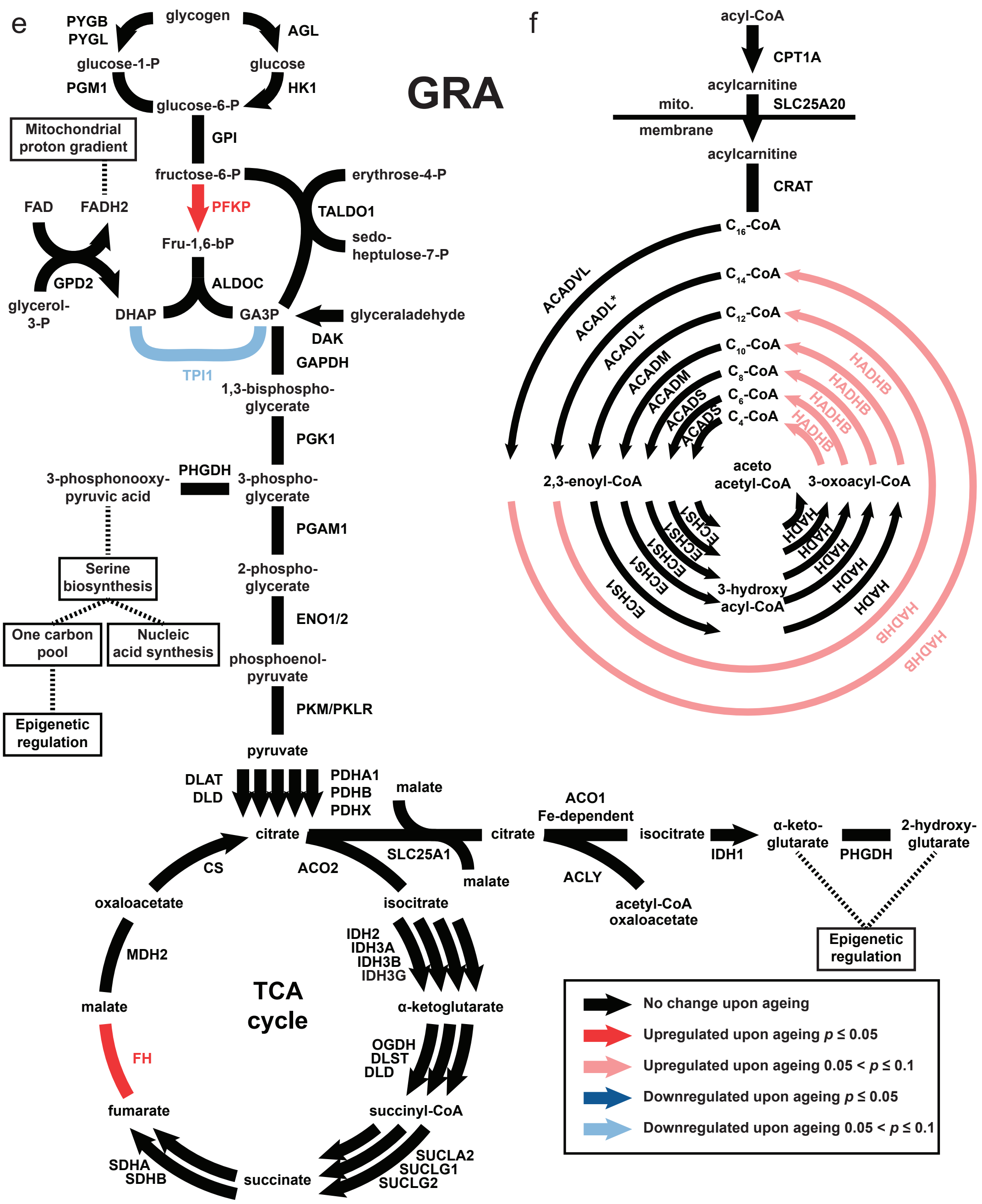

Supplementary Figure 11 e-f: Prominent changes upon ageing in the central carbon metabolism of GRA. (e) Glucose metabolism and the tricarboxylic acid (TCA) cycle are illustrated with arrows representing unidirectional reactions and strokes representing bidirectional reactions. The gene names of the respective enzymes are written in capital letters and the colour encodes changes upon ageing, as described in the legend. (f) The mitochondrial beta-oxidation of saturated fatty acids is depicted as described for (e). A star indicates that the particular protein was not covered for quantification. All *p*-values (p) are based on Spearman correlation analyses.

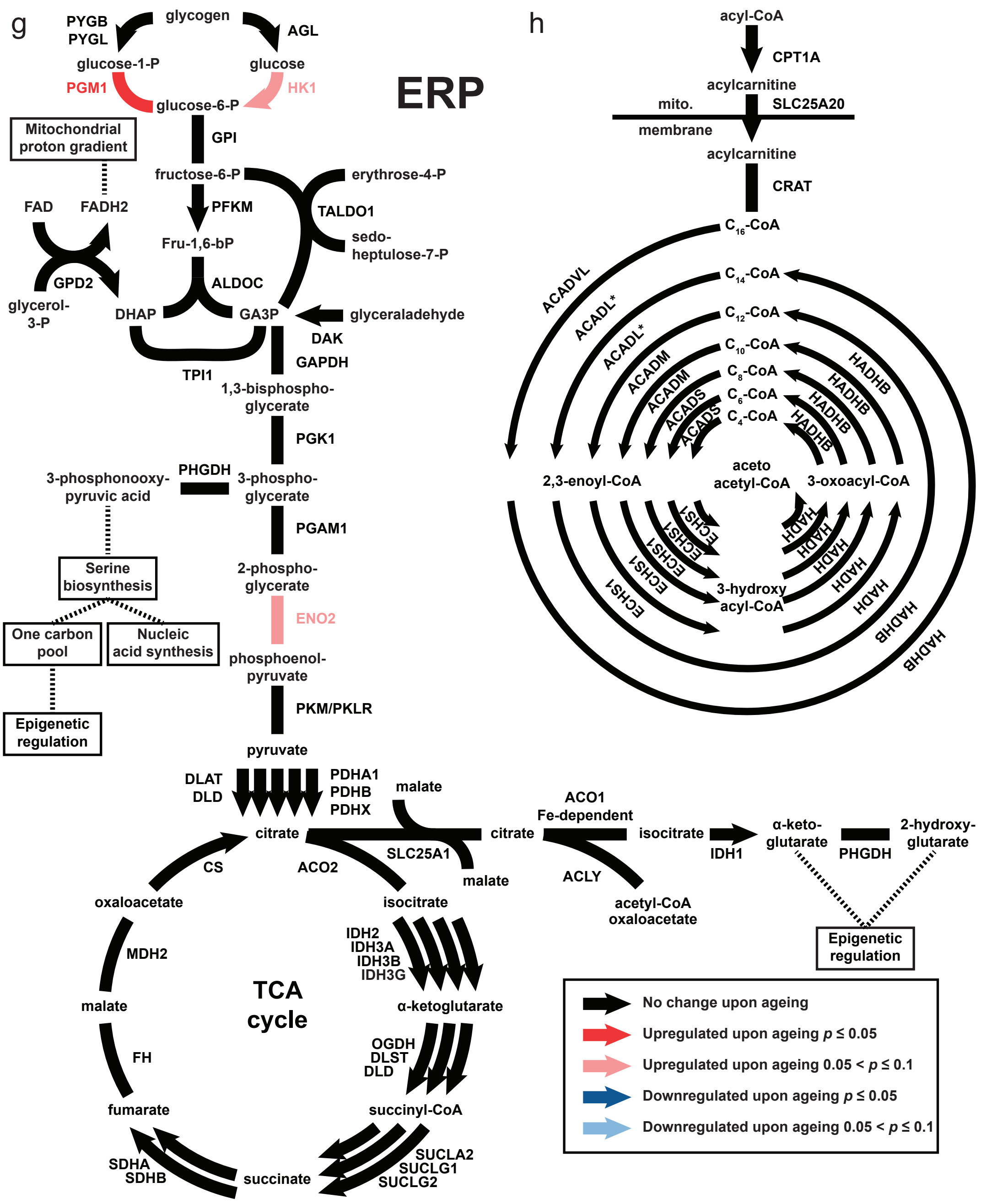

Supplementary Figure 11 g-h: Prominent changes upon ageing in the central carbon metabolism of ERP. (g) Glucose metabolism and the tricarboxylic acid (TCA) cycle are illustrated with arrows representing unidirectional reactions and strokes representing bidirectional reactions. The gene names of the respective enzymes are written in capital letters and the colour encodes changes upon ageing, as described in the legend. (h) The mitochondrial beta-oxidation of saturated fatty acids is depicted as described for (g). A star indicates that the particular protein was not covered for quantification. All *p*-values (*p*) are based on Spearman correlation analyses.

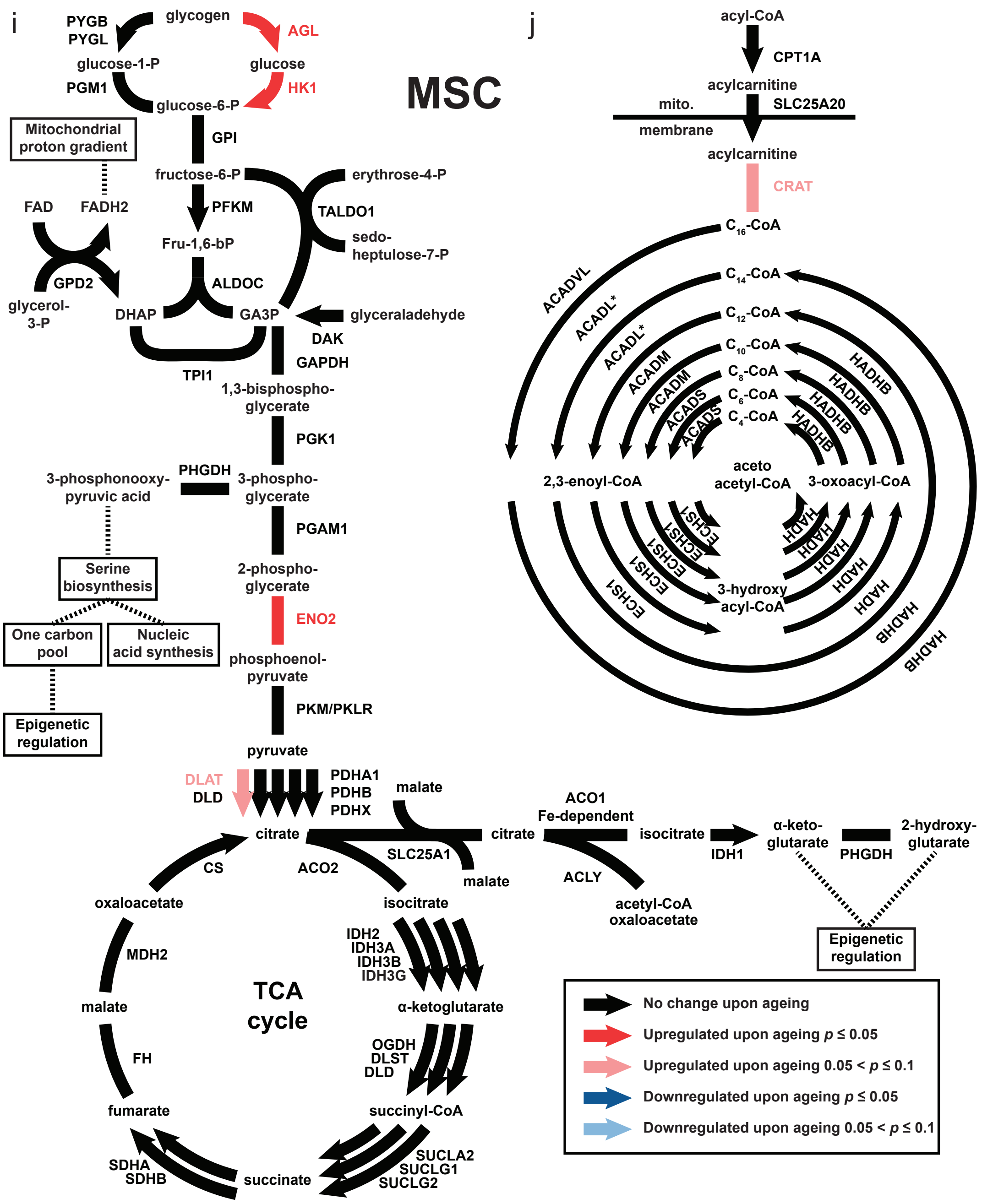

Supplementary Figure 11 i-j: Prominent changes upon ageing in the central carbon metabolism of MSC. (i) Glucose metabolism and the tricarboxylic acid (TCA) cycle are illustrated with arrows representing unidirectional reactions and strokes representing bidirectional reactions. The gene names of the respective enzymes are written in capital letters and the colour encodes changes upon ageing, as described in the legend. (j) The mitochondrial beta-oxidation of saturated fatty acids is depicted as described for (i). A star indicates that the particular protein was not covered for quantification. All *p*-values (*p*) are based on Spearman correlation analyses.

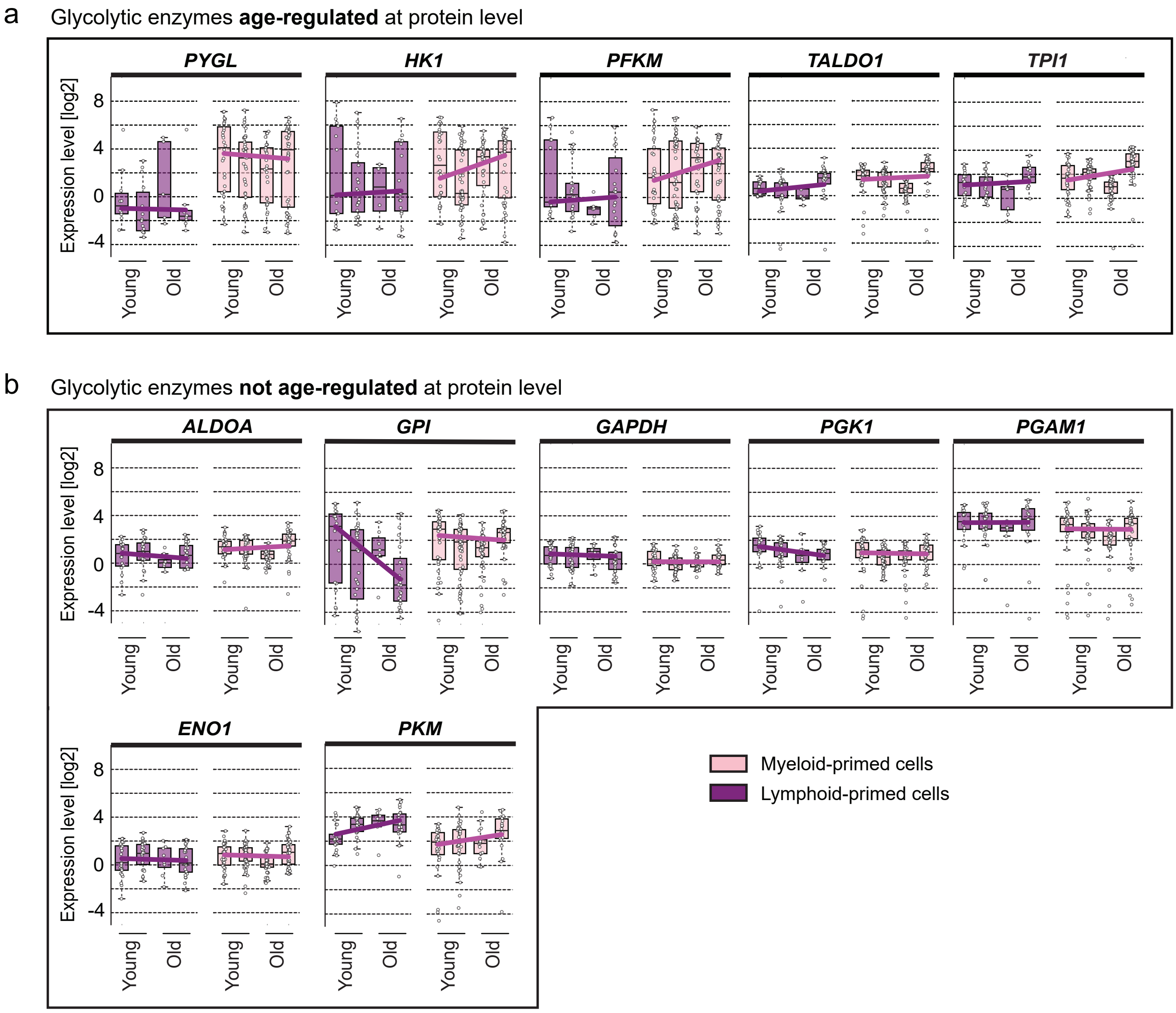

Supplementary Figure 12. Single-cell analysis reveals lineage- and age-dependent increase of glycolytic enzymes. Box-plots representing expression values of a gene in lymphoid- primed (purple) and myeloid-primed (pink) cells in a given sample (2 young (S4+S2), and 2 old (S3+S1)). The central line in the box plots indicates the median, the bottom and top edges of the box the IQR, and the box plot whiskers represent 1.5 times the IQR. A trend line connects the respective box-plot medians; the respective slope is further used for Figure 7b. (a) Box-plots representing genes of the preparatory phase of glycolysis that were found to be age-regulated (*p*-value < 0.05, Spearman correlation), and quantified in the single-cell RNA-seq data. (b) Box-plots representing genes of glycolysis that were found to be not age-regulated (*p*-value > 0.05, Spearman correlation), and quantified in the single-cell RNA-seq data.

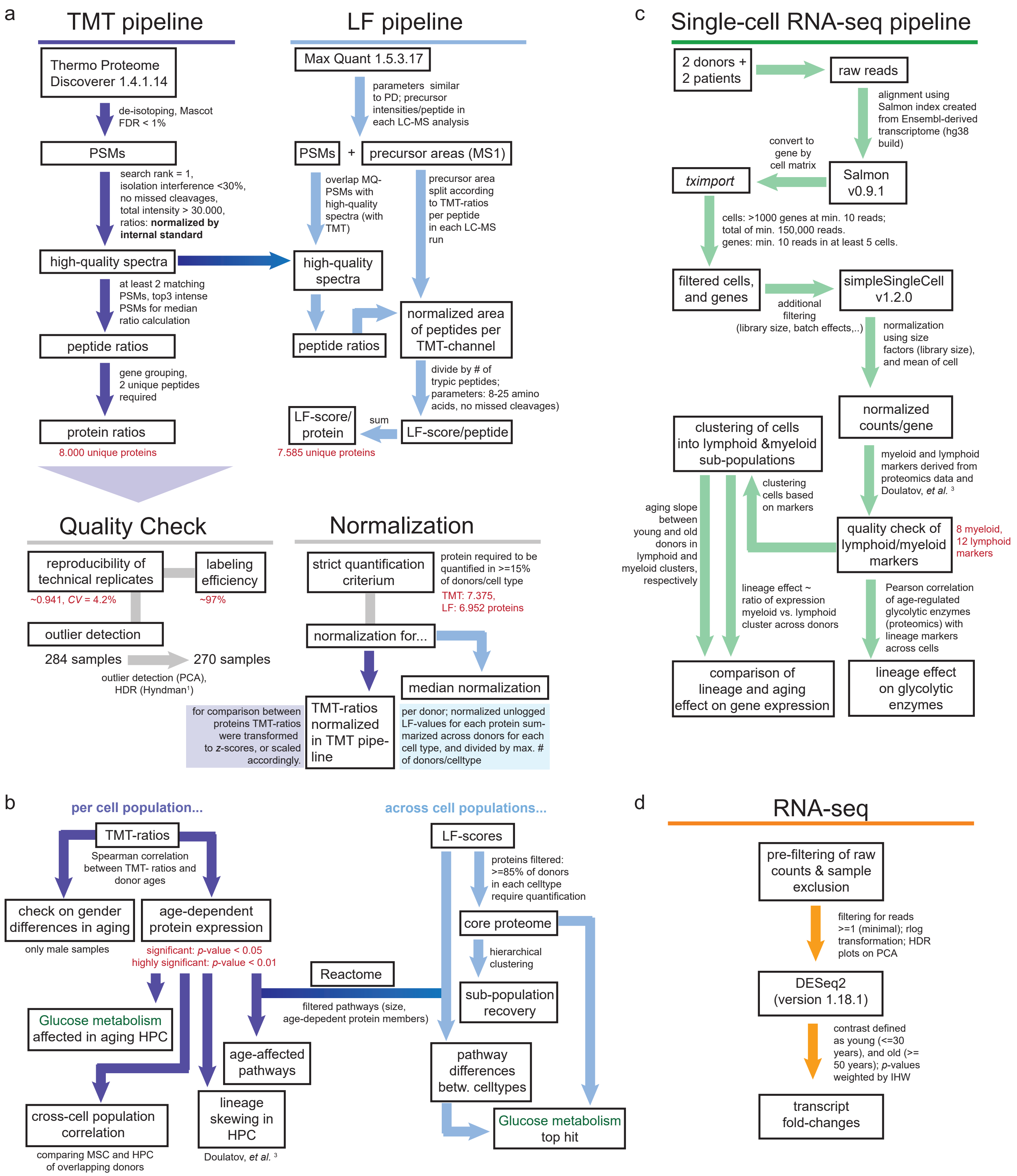

Supplementary Figure 13. Overview on the computational data processing. (a) Delineation of the processing pipeline after acquisition of mass-spectrometry data. (b) Data-processing after obtaining TMT-ratios and LF-scores in (a). (c) Single-cell RNA-seq processing pipeline after acquisition of raw reads. (d) Bulk transcriptome analysis with DESeq2, as explained in Methods.

Supplementary Tables

Supplementary Table 1: Overview of all clinical samples processed for the proteomics and transcriptomics data.

Proteomics

Age	Sex	FACS purity [%] and availability					MSC	#samples per donor
		HPC	LYM	MON	GRA	ERP		
36	m	94.5	98.8	87.8	98.0			4
24	m	97.0	99.0	96.4	97.6	100.0		5
33	m	98.7	99.3	96.0	98.0	100.0	100	6
46	f	100.0	100.0	98.7	98.0	100.0		5
50	m	98.4	97.2	97.0	97.6	93.9	100	6
20	m	97.6	99.0	97.8	97.4	96.2	100	6
27	m		99.6			98.2	100	3
45	m						100	1
32	m	98.4	98.8			98.4	100	4
25	f		99.6	94.3		97.2	100	4
37	m	92.5	96.0	95.5	96.5	N/A	100	6
48	m		97.3			99.7	100	3
21	m	95.0	97.5	95.5	98.0	97.5	100	6
29	m						100	1
23	m	93.9	95.3	83.1	97.5	99.2	100	6
21	m	98.0	97.8	99.2	98.2	98.2		5
48	f		97.5			100.0		2
57	f		96.5		95.0	99.0		3
54	m		98.2		96.0	99.2		3
37	m		96.2	95.6	96.3	98.5	100	5
57	m	90.3	99.5	96.6	96.5	95.5	100	6
39	m	92.3	98.6	98.7	95.5	98.1	100	6
31	f		99.4	74.5	95.5	100.0	100	5
39	f	95.0	99.0	97.9	98.0	96.9		5
24	m	99.0	96.4	98.5	96.0	99.0		5
21	m	94.8	99.0	97.4	98.0	98.4		5
53	m						100	1
55	m	90.5	97.0	92.0	93.3		100	5
30	m		98.0	98.0	97.2	95.5	100	5
50	m	95.4	96.7	78.7	97.7	98.8	100	6
25	m		98.1	93.9	98.5	99.5	100	5
31	f	94.4	94.0	86.7	89.8	96.7	100	6
32	m	90.7	97.9	95.7	96.0	96.4	100	6
30	m	N/A	N/A	N/A	N/A	N/A	100	6
39	m	95.0	99.1	98.1	97.1	100.0	100	6
28	f	94.0	98.9	84.8	87.4	98.1	100	6
48	f	79.4	98.9	86.6	94.9	97.6		5
22	f	89.2	64.9	97.5	95.6	96.9	100	6
59	f						100	1
48	m		97.4	97.6	96.0	97.6	100	5
21	m	92.9	96.6	93.4	89.8	98.6	100	6
33	m	92.6	96.0	89.4	95.4	98.6	100	6
56	m	96.6	91.7	85.0	93.2	96.7		5
56	f		99.0	91.2	95.5	99.1	100	5
41	m		98.6	98.0	94.6	99.5	100	5
29	f	N/A	N/A	N/A	N/A	N/A	100	6
30	m	97.5	97.1	88.1	92.6	99.5	100	6
27	m	96.5		98.0	97.5	99.0	100	5
29	m	98.0	91.9	83.2	98.5	96.0	100	6
39	m	98.5	100.0	96.1	97.0	100.0		5
28	m	93.7	94.5	90.3	87.9	91.5	100	6
60	m	98.1		96.9		98.4	100	4
29	m						100	1
41	m	93.4	97.6	98.0	98.7	99.4		5
57	m	97.1	99.8	99.2	97.4	99.2	100	6
41	f		98.8	98.2	98.6	99.0	100	5
44	m						100	1
23	m						100	1
29	m						100	1
#samples		37	49	45	45	49	45	270

donors total:	59	sample in final dataset
male donors:	45	outlier
female donors:	14	sample not used or available

Transcriptomics

Age	Sex	FACS purity [%] and availability					MSC	#samples per donor
		HPC	LYM	MON	GRA	ERP		
20	m		99.0	97.8	97.4	96.2	100	
25	f	94.9	99.6	94.3	91.0	97.2	100	6
57	f	85.7	96.5	97.5	95.0	99.0		5
54	m	86.2	98.2	96.1	96.0	99.2		5
57	m		99.5	96.6		95.5	100	4
55	m	90.5	97.0	92.0			100	4
50	m	95.4	96.7	78.7	97.7	98.8	100	6
28	f	94.0	98.9		87.4	98.1	100	5
22	f	89.2	64.9	97.5	95.6	96.9	100	6
56	m	96.6	91.7	85.0		96.7		4
56	f	81.0	99.0	91.2	95.5	99.1		5
30	m	97.5	97.1	88.1	92.6	99.5		5
60	m	98.1	96.2	96.9		98.4	100	5
#samples		11	13	12	9	12	8	65

donors total:	13	sample in final dataset
male donors:	8	outlier
female donors:	5	sample not used or available

Overview of the age, gender, available cell populations and purity of the FACS for all 59 donors of the proteomics and transcriptomics study.

Supplementary Table 2: Overview of all clinical samples processed for the metabolomics data and single cell transcriptomics data

Age	Sex	FACS purity [%] of HPC	Metabolomics	Single cell RNA-seq
63	m	94	x	
59	m	92	x	x
69	m	88	x	
31	f	97	x	x
62	f	92	x	x
52	m	91	x	
44	m	94	x	
23	m	93	x	
21	f	89	x	x
33	f	75	x	

x sample in final dataset
sample not used or available

Supplementary Table 3: Overall numbers for single cell transcriptomics

Sample ID	meta-data	# of cells retained	% of cells retained	median library size	# of unique genes	average # of genes/cell	# of lymphoid cells (fraction of all cells)	# of myeloid cells (fraction of all cells)
1	male/59.2 years old	101	52.6%	686,990	11,976	5,819	9 (8.9%)	49 (48.5%)
2	female/30.6 years old	152	79.2%	1,662,115	13,374	5,711	35 (23.0%)	58 (38.2%)
3	female/62.2 years old	127	66.1%	1,656,706	13,093	5,991	32 (25.2%)	48 (37.8%)
4	female/21.2 years old	139	72.4%	1,212,586	12,693	5,274	28 (20.1%)	41 (29.5%)

The columns list the age and gender of the donor, the number of cells analyzed per donor (# of cells retained), the median library size, the number of unique genes, the average number of genes per cell, as well as the fraction of lymphoid- and myeloid-primed cells per donor.

Supplementary References

1. Hyndman RJ. Computing and graphing highest density regions. *Am Stat* **50**, 120-126 (1996).
2. Uhlen M, *et al.* Tissue-based map of the human proteome. *Science* **347**, doi: 10.1126/science.1260419 (2015).
3. Doulatov S, Notta F, Eppert K, Nguyen LT, Ohashi PS, Dick JE. Revised map of the human progenitor hierarchy shows the origin of macrophages and dendritic cells in early lymphoid development. *Nat Immunol* **11**, 585-593 (2010).
