# Supplementary material for: Cell-specific proteome analyses of human bone marrow reveal molecular features of age-dependent functional decline
Source: Nat Commun. 2018 Oct 1;9:4004. doi: 10.1038/s41467-018-06353-4 (PMC6167374; doi:10.1038/s41467-018-06353-4)
Supplement: Supplementary file 3 — Description of Additional Supplementary Files [file 41467_2018_6353_MOESM3_ESM.docx]

**Description of Additional Supplementary Files**

File Name: Supplementary Data 1

Description: List of all proteins identified by LC-MS/MS. The columns delineate the cell population, gene name, gene description, ensemble and uniprot identifiers of the proteins with the respective gene name and of which peptides have been identified, number of donors in which the protein has been observed, number of proteins with the respective gene name of which peptides have been identified, and the total number of unique peptides and peptide spectrum matches (PSMs).

File Name: Supplementary Data 2

Description: List of all proteins quantified by label-free quantification. The columns define the gene name, the gene description, the uniprot protein identifiers, the number of samples the protein has been quantified in, the number of donors the gene has been identified in per individual cell population, an annotation if the protein is specific to the cell population or part of the core proteome and 6 columns for the normalized label-free quantification values for each cell population.

File Name: Supplementary Data 3

Description: List of pathways with different stoichiometry in the different cell populations. The list contains the underlying data of Figure 2c, specifying the mean abundance of Reactome-derived pathways and the fraction in % that is changing in stoichiometry across the different cell populations (*p*-value included as well, Wilcoxon-test).

File Name: Supplementary Data 4

Description: List of proteins quantified by TMT. The columns define the cell population, the gene name and the gene description, the number of proteins per gene that contain an identified peptide, the uniprot and ensemble identifiers of these proteins, the number of samples the protein has been quantified in, the total number of unique peptides of the protein, the Spearman correlation coefficient and Spearman *p*-value calculated based on the z-score normalized data, and the slope calculated based on the average normalized data.

File Name: Supplementary Data 5

Description: List of all transcripts quantified by RNAseq. List containing bulk RNA-seq data across different donors and cell populations. The list shows the transcript fold-changes (with respective *p*-values) between young (50 years) donors for each quantified transcript in each cell population. For comparison purposes it also contains the Spearman correlation coefficient and *p*-value from Supplementary Data File 4.

File Name: Supplementary Data 6

Description: Summary of all numbers relevant to the study. The number of proteins quantified by TMT and label-free (LF) quantification that are covered in more or equal to 85%, more than 15%, or at least a single (>0%) of all donors are listed. The gene names and numbers of cell population specific proteins defined as being quantified in more or equal to 85% of all donors and being quantified in less than 15% of all donors in each other cell population are listed. The numbers of proteins being quantified by TMT or LF quantification in more than a single cell population and classified by the number of cell populations are reported. The numbers of proteins altered upon ageing at p- values < 0.05 and < 0.01 (Spearman correlation) in the different cell populations at different coverages and subdivided into up- or downregulated are mentioned as well.

File Name: Supplementary Data 7

Description: List of pathways that contain proteins that are significantly (*p*-value < 0.05, Spearman correlation) altered upon ageing. The list contains as columns the cell population, Reactome pathway identification, pathway name and hierarchy, proteins associated with the pathway in the Reactome database, gene names of the proteins quantified in the respective cell population, number of proteins quantified in the respective cell population, number of proteins quantified in all cell populations, percentage of the ratio between the number of proteins quantified in the respective cell population and all proteins of the pathway, number of proteins significantly (*p*-value < 0.05, Spearman correlation) altered, percentage of the ratio between the number of proteins significantly altered and the number of proteins quantified in the respective cell population, average slope of the altered proteins, gene names of the upregulated and downregulated proteins and the level1 of the Reactome database to which the pathway is assigned.

File Name: Supplementary Data 8

Description: Metabolomic data of HPCs originating from ten human subjects. The table includes results for the calibration curves and quality control (QC) measurements. For each metabolite and experiment, the following is listed: the expected amount, calculated amount, and % difference between expected and calculated amount for the calibration curve and the QCs as well as the calculated amount for the HPC measurements. The linear calibration curves had a 1/X weighting and their equation and *R^2^* are listed.

File Name: Supplementary Software

Description: The file contains all code that was specifically written for the data analyses.
